# Cascade AOA Estimation Algorithm Based on Flexible Massive Antenna Array

**DOI:** 10.3390/s20236797

**Published:** 2020-11-28

**Authors:** Tae-yun Kim, Suk-seung Hwang

**Affiliations:** 1 Department of Electronic Engineering, Chosun University, Gwangju 61452, Korea; skriekd12@chosun.kr; 2 School of Electronic Engineering, Chosun University, Gwangju 61452, Korea

**Keywords:** Angle-of-Arrival (AOA), cascade estimation, CAPON, Beamspace MUSIC, flexible massive antenna

## Abstract

The Angle-of-Arrival (AOA) has a variety of applications in civilian and military wireless communication fields. Due to the rapid development of the location-based service (LBS) industry, the importance of the AOA estimation technique has increased. Although a large antenna array is necessary to estimate accurate AOA information of many signals, the computational complexity of conventional AOA estimation algorithms, such as Multiple Signal Classification (MUSIC), is dramatically increased. In this paper, we propose a cascade AOA estimation algorithm employing CAPON and Beamspace MUSIC, based on a flexible (on/off) antenna array. First, this approach roughly finds AOA groups, including several signal AOAs using CAPON, by applying some of the antenna elements. Then, it estimates each signal AOA in the estimated AOA groups using Beamspace MUSIC by applying the full size of the antenna array. In addition to extremely low computational complexity, the proposed algorithm also has similar estimation performance to that of MUSIC. In particular, the proposed cascade AOA estimation algorithm is highly efficient when employing a massive antenna array. Representative computer simulation examples are provided to illustrate the AOA estimation performance of the proposed technique.

## 1. Introduction

Angle-of-Arrival (AOA estimation) is a core technique in wireless communication systems employing a location detection technology (LDT), and has various applications ranging from commerce to the military. The AOA information of a signal is generally estimated by an array antenna installed in various communication systems, such as a radar receiver and a satellite [1,2,3,4,5,6]. The structure of an array antenna utilized to estimate signal AOAs varies, and includes the linear array, planar arrays (L-shape, square, circular, hexagonal, etc.), and arbitrary array antennas [7,8,9], which can be combined in several shapes [10,11].

AOA estimation techniques are mainly classified into classical, subspace-based, and maximum likelihood (ML) methods [8,12]. Although classical methods, such as the Bartlett and CAPON algorithms, are simply implemented because of their low complexity, they do not have good estimation performance in general. Representative subspace-based methods, based on the orthogonal property between signal and noise subspaces, are represented by the MUSIC and Estimation of Signal Parameter via Rotational Invariance Techniques (ESPRIT) algorithms. Although these have higher computational complexity than the classical method, their estimation performance is usually much better than that of the classical method. ML techniques, such as the Alternating Projection Algorithm (APA) [7,8,9,13,14], have the best estimation performance, but generally have the highest computational complexity.

Various studies evaluating and comparing performances of AOA estimation algorithms exist. AOA estimation performance, based on Uniform Linear Array (ULA), Bartlett, Minimum Variance Distortionless Response (MVDR), linear prediction approach, and MUSIC were compared and analyzed in [15]. In addition, the study evaluated the resolution sensitivity due to changing parameters, such as the number of antenna array elements, for each AOA estimation algorithm. Modified ULA configurations with higher resolution to improve the AOA estimation performance of CAPON and MUSIC algorithms were presented in [10]. Based on ULA with four antenna elements, the estimation performance of MUSIC and ESPRIT algorithms, which are commonly employed in smart antenna systems, were compared and analyzed in [16]. Results showed that the estimation performance of MUSIC was more accurate than that of ESPRIT.

Based on ULA, the estimation accuracy and computational complexity of MUSIC, Root-MUSIC, and ESPRIT algorithms were evaluated and analyzed with performance evaluation scenarios, including changes in the number of antenna elements versus the signal-to-noise ratio (SNR), in [17]. A modified MUSIC algorithm using condensate data, which has better estimation performance than the conventional MUSIC algorithm, confirmed by simulation results based on ULA, uniform rectangular array (URA), and uniform circular array (UCA) antenna structures, was proposed in [18]. In addition, performances of CAPON and MUSIC algorithms based on UCA, for the relationship between mean square error (MSE) and system resolution, were compared and analyzed in [19]. A uniform representation of ESPRIT and Min-Norm algorithms, based on data parameters such as the number of snapshots, and the consistency and the separation factor of sources, was derived, and the influence of the parameters on estimation error was analyzed in [20].

A signal subspace scaled MUSIC (SSMUSIC) proposed in [21] estimates the signal AOA based on the linear algebraic connection between the standard subspace of the correlation matrix and the subspace of the specific signal plus an interference model. Subspace-based algorithms were classified into three methods by mathematical procedures, namely, an extrema-searching approach including the MUSIC and Min-Norm searching algorithms, a polynomial-rooting approach including Min-Norm, and Root-MUSIC, and a matrix-shifting approach including State-Space Realization, ESPRIT, and Matrix-Pencil method, in [22]. The Root-MUSIC algorithm was described and its superior estimation performance for the ULA structure compared to the conventional MUSIC algorithm was verified in [23]. In addition, the performance evaluation and analysis of Bartlett, CAPON, MUSIC, and ESPRIT algorithms for adjacent signal sources, based on ULA, were provided in [24].

To accurately estimate AOAs for adjacent signal sources, we should employ an antenna array with multiple elements [25]. To resolve the high complexity problem of the conventional AOA estimation algorithm for large antenna arrays, [26] proposed the compressed MUSIC (C-MUSIC) approach, which search signal AOAs in a small angular sector, similar to the Beamspace MUSIC [27] or sector-focused approaches [28]. In addition, a two-dimensional AOA algorithm with reduced complexity based on the unitary ESPRIT algorithm was introduced in [25], and an efficient AOA estimation technique for a massive ULA, using fast Fourier transform (FFT), was proposed in [29].

Most of the studies of AOA estimation have been carried out with a general antenna structure with a small size rather than a large antenna structure. However, because the demand for massive antennas has increased with the development of technology, including wireless communication techniques, recently, studies for AOA estimation based on massive antennas have been conducted. The complexity involved in estimating the signal AOA based on a massive antenna array may increase exponentially, which is a significant problem in applications such as radar, satellite, and telecommunication devices, because they need to estimate the AOA in real time. Various techniques for reducing the complexity of AOA estimation based on a massive multiple-input multiple-output (MIMO) system were presented in [4,30,31,32,33]. The most effective means of reducing the complexity of the AOA estimation algorithm is to reduce the dimension of the antenna array, like in the Beamspace method [7,8,13,34].

The main goal of this paper is to address the issue of the high computational complexity of massive antenna arrays to accurately estimate multiple signal AOAs. Although the performance of the AOA estimation algorithm is enhanced for a larger antenna array, its computational complexity may be dramatically increased. As a result, the computational complexity involved in estimating AOA based on a massive antenna array is extremely high. To address this high complexity problem for estimating the signal AOA, in this paper, we propose a cascade AOA estimation algorithm based on a flexible massive antenna array, consisting of CAPON and Beamspace MUSIC algorithms. We define a flexible massive antenna as comprising array elements with an on/off function. The proposed cascade algorithm is distinguished by two steps:

1. Step 1: Find searching ranges employing CAPON, with a small number (small proportion) of array elements.
2. Step 2: Estimate detail AOAs in the estimated ranges employing Beamspace MUSIC, with the entire number of array elements.

In the proposed cascade algorithm, the goal of utilizing a small number of array elements with CAPON is to roughly estimate the ranges including signal AOAs. The goal of utilizing the entire number of array elements with Beamspace MUSIC is to estimate individual signal AOAs in the ranges estimated by CAPON. Because this cascade approach does not search the entire range to estimate the detailed signal AOAs, it is highly efficient and fast compared to conventional AOA estimation algorithms, such as MUSIC, for a massive antenna array.

The remainder of this paper is organized as follows. Section 2 describes the received signal model, which includes the signal, antenna array vector, and additive white Gaussian noise (AWGN). We present the cascade AOA estimation algorithm based on a flexible massive antenna, consisting of CAPON and Beamspace MUSIC, in Section 3. In Section 4, we discuss the computational complexity of the proposed approach and compare it to the conventional AOA estimation algorithm. Computer simulation results are provided to illustrate the AOA estimation performance of the proposed approach in Section 5. Finally, Section 6 outlines the conclusions of this study.

## 2. Received Signal Model

In this section, we describe the received signal model including multiple signals, mathematical antenna array model, and AWGN.

### 2.1. Signal Model

Assuming that L signals are incident on the antenna array with the size of M×N(P=MN antenna elements), the received signal vector at discrete sample index k can be modeled as:

(1) r(k)=As(k)+n(k)

where A is the P×L array response matrix, $s\left(k\right)$ is the signal vector (size L), and $n\left(k\right)$ is the AWGN vector (size P) with independent and identically distributed (i.i.d.) components, each of which has zero mean and variance $\sigma^{2}$.

### 2.2. Flexible Massive Antenna Model

The array response matrix A in Equation (1) is defined as:

(2) $$A≜\left[\begin{array}{ccc} 1 & \cdots & 1\\ e^{-j\mu_{1}} & \cdots & e^{-j\mu_{L}}\\ \vdots & \ddots & \vdots \\ e^{-j(M-1)\mu_{1}} & \cdots & e^{-j(M-1)\mu_{L}}\\ e^{-j\eta_{1}} & \cdots & e^{-j\eta_{L}}\\ e^{-j(\mu_{1}+\eta_{1})} & \cdots & e^{-j(\mu_{L}+\eta_{L})}\\ \vdots & \ddots & \vdots \\ e^{-j((M-1)\mu_{1}+(N-1)\eta_{1})} & \cdots & e^{-j((M-1)\mu_{L}+(N-1)\eta_{L})} \end{array}\right]$$

where

(3) $$\mu_{l}=2\pi (d/\lambda )\sin\theta_{l}\cos\phi_{l}$$

(4) $$\eta_{l}=2\pi (d/\lambda )\sin\theta_{l}\sin\phi_{l}$$

In Equations (3) and (4), $\theta_{l}$ and $\phi_{l}$ are the elevation angle and the azimuth angle, respectively, d is interelement spacing, and λ is the wavelength of the signals.

**Figure 1:**
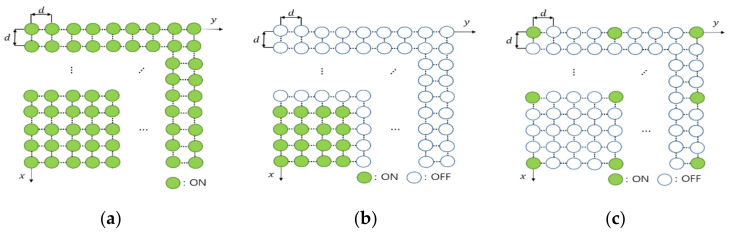
Examples of the flexible antenna array: (**a**) turning on entire antenna elements, (**b**) turning on some concentrated array elements, (**c**) turning on some scattered array elements.

In this paper, we consider a flexible massive antenna array, which is defined as one in which some of the antenna elements can be turned on and some of them can be turned off, depending on the situation, as shown in Figure 1. Figure 1 describes three examples of the flexible massive antenna array; all array elements are turned on in Figure 1a, some concentrated array elements are turned on in Figure 1b, and some scattered array elements are turned on in Figure 1c.

## 3. Cascade AOA Estimation Algorithm Based on Flexible Massive Antenna Array

**Figure 2:**
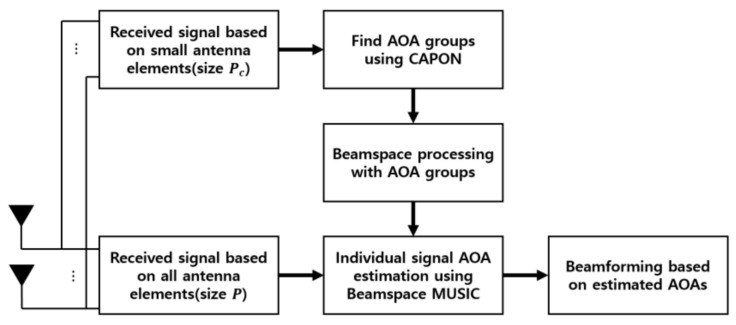
Architecture of cascade Angle-of-Arrival (AOA) estimation algorithm based on flexible (ON/OFF) massive antenna array.

In the use of large antenna arrays to estimate the signal AOA, the estimation performance of conventional AOA estimation techniques, such as MUSIC, is significantly enhanced, but its computational complexity is dramatically increased. To address the problem of extremely high computational complexity and efficiently estimate signal AOAs based on a massive antenna array, in this section we propose a cascade AOA estimation algorithm consisting of CAPON and Beamspace MUSIC, as shown in Figure 2.

First, we roughly find AOA groups, including multiple signal AOAs for estimating the ranges of the groups, using CAPON. In this process, we use a proportion of the array elements (small size) to roughly estimate their ranges. Next, we estimate the detailed signal AOAs in the estimated AOA groups, using Beamspace MUSIC. In this process, we use all of the array elements (full size) to enable accurate estimation of each AOA. To estimate the detailed AOAs, we scan only the estimated ranges, unlike in conventional AOA estimation techniques, such as MUSIC. In the proposed cascade AOA estimation algorithm, the goals of CAPON and Beamspace MUSIC are to roughly estimate the ranges of existing signal AOAs and to estimate individual signal AOAs in ranges estimated by CAPON, respectively.

### 3.1. CAPON for Estimating AOA Range Based on Small Number of Antenna Element

The CAPON algorithm is designed to estimate signal AOAs using the property that maximizes the output SNR for the incident signal to the antenna [35]. Although CAPON has poor resolution for estimating AOAs of adjacent signals or in a low SNR environment, it has low computational complexity and fast estimation performance compared to a subspace-based algorithm that requires eigenvalue decomposition [3,36].

The proposed cascade algorithm in this paper quickly and roughly identifies AOA groups, including multiple adjacent AOA signals, using CAPON. In this process, it utilizes a small number of the total antenna elements because we need to merely find approximate AOA groups. The spatial spectrum of CAPON for identifying the AOA group is given by:

(5) $$P_{CAPON}=\frac{1}{a{\left(\theta ,\phi \right)}^{H}R_{C}^{-1}a\left(\theta ,\phi \right)}$$

where $R_{C}$ is a covariance matrix for the received signal based on the small number of antenna elements, defined as:

(6) $$R_{C}=E\left[r_{C}\left(k\right)r_{C}{\left(k\right)}^{H}\right]$$

In Equations (5) and (6), $a\left(\theta ,\phi \right)$ is an array response vector for the specific elevation and azimuth angles, $r_{C}\left(k\right)$ is the received signal based on the small number of antenna elements,$P_{C}=M_{C}\times N_{C}$ ($M_{C}$ and $N_{C}$ are the numbers of antenna elements turned on in each axis), and ${}^{H}$ is the conjugate transpose. Using peaks of Equation (5), we roughly find AOA groups including multiple signal AOAs, and determine the ranges of existing signal AOAs based on the estimated AOA groups.

### 3.2. Beamspace MUSIC for Estimating Signal AOA Based on Entire Antenna Element

Although the estimation performance of the adjacent signal AOAs is enhanced as the number of antenna elements is increased, the computational complexity of estimation processing is dramatically increased [37]. Therefore, a technique for minimizing performance degradation and reducing the dimension of an antenna array with large number of elements is required to ensure high estimation performance of adjacent signal AOAs. The representative method to reduce the antenna array dimension is a linear transformation technique. Beamspace processing reduces the antenna dimension from an element space to a beamspace using linear transformation. The dimensional reduction by beamspace processing is performed by multiplying the conjugate transpose of the linear transformation matrix with size of $P\times P_{B}$ (typically $P_{B}\ll P$), where P is the number of antenna elements and $P_{B}$ is the dimension of the beamspace, by the received signal vector. To cover the entire array element dimension, multiple beamspace transformation matrices may be considered. Algorithms based on beamspace processing, such as Beamspace MUSIC [38], Beamspace root MUSIC [39], and Beamspace ESPRIT [40], have several benefits, including reduced computational complexity, improved resolution, and reduced estimation error [3,5,6,7,27,41]. The estimation performance of the Beamspace MUSIC algorithms can approach the Cramer–Rao Bound (CRB) if appropriate preprocessor settings are used [42].

To estimate the detailed signal AOAs included in the estimated AOA groups, the proposed cascade algorithm employs Beamspace MUSIC. Unlike the process of CAPON, which uses a small proportion of the total number of antenna elements, Beamspace MUSIC uses all of the antenna elements (full size of the antenna array) in the estimated ranges of AOA groups. That is, an AOA group obtained by CAPON becomes a beam sector (range for searching) and Beamspace MUSIC finds the detailed AOA of each signal in that beam sector.

The beamspace transformation matrix can be generated by the discrete Fourier transform (DFT), discrete prolate spheroidal sequence (DPSS), or Taylor series [8,43]; we consider the DFT method in this paper. The beamspace transformation matrix applied to the full-size antenna array is defined as:

(7) $$B≜\frac{1}{\sqrt{P}}B_{y}⊗B_{x}$$

where $B_{y}$ and $B_{x}$ represent DFT metrics for the x and y axes, respectively, and ⊗ is a Kronecker operator. In Equation (7), the size of B is $P\times P_{B}$. The beamspace output vector is given by:

(8) $$q\left(k\right)=B^{H}r\left(k\right)$$

where $r\left(k\right)$ is the received signal vector based on all of the antenna array elements. The spatial spectrum of Beamspace MUSIC is given by:

(9) $$P_{BeamspaceMUSIC}\left(\theta ,\phi \right)=\frac{a{\left(\theta ,\phi \right)}^{H}BB^{H}a\left(\theta ,\phi \right)}{a{\left(\theta ,\phi \right)}^{H}BE_{BN}E_{BN}^{H}B^{H}a\left(\theta ,\phi \right)}$$

where $E_{BN}$ is a beamspace noise subspace eigenvector matrix calculated by the eigenvalue decomposition of the beamspace covariance matrix $R_{B}$, which is defined as:

(10) $$R_{B}=E\left[q(k)q{\left(k\right)}^{H}\right]$$

Using the peaks of Equation (9), we estimate the detailed signal AOAs included in AOA groups. Because the proposed cascade algorithm only searches for signal AOAs in specific sectors, its computational complexity is substantially lower than that of conventional AOA estimation algorithms, such as MUSIC, which searches for AOAs across the entire range of angles for the massive array antenna.

**Figure 3:**
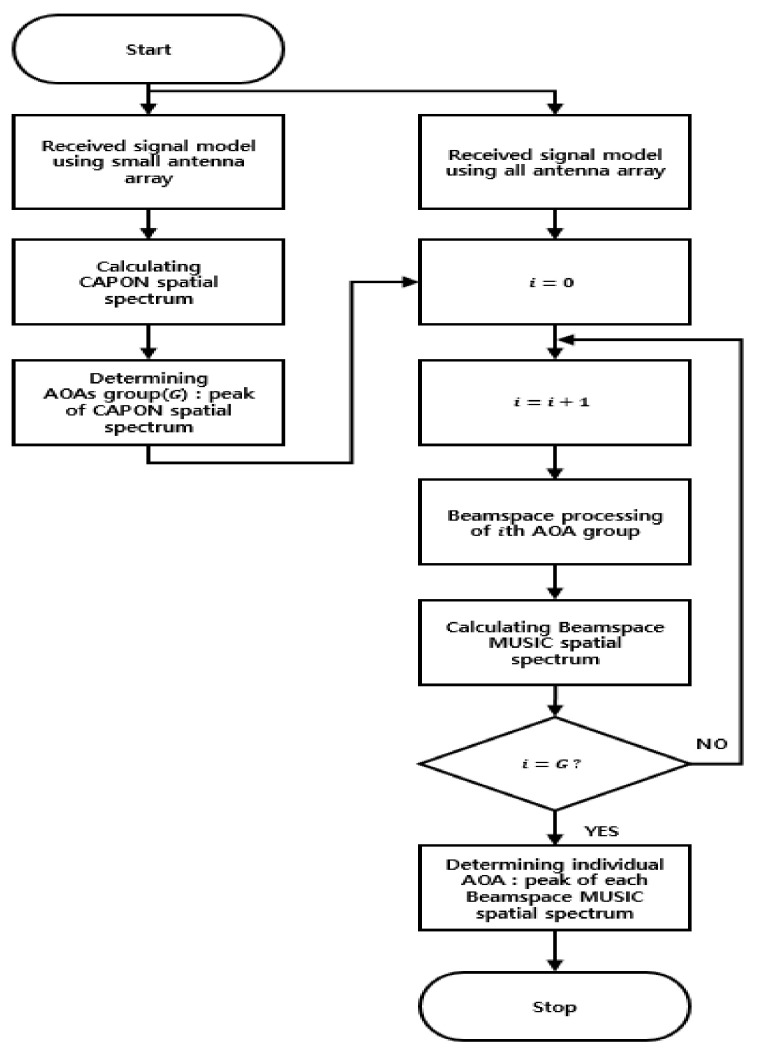
Flow chart of the proposed cascade algorithm.

The proposed cascade AOA estimation algorithm based on the flexible massive antenna is summarized in Table 1 and Figure 3. Although a massive antenna array has excellent performance for estimating accurate AOAs of multiple signals simultaneously, it suffers from extremely high computational complexity due to the size of the antenna array. To address the issue of high computational complexity in the estimation of signal AOAs based on a massive antenna array, in this paper we propose the cascade AOA estimation algorithm consisting of CAPON and Beamspace MUSIC. To reduce the computational complexity, we select searching ranges including signal AOAs using CAPON, and we estimate the detailed AOAs using Beamspace MUSIC only in the selected ranges. Because CAPON uses a small number of antenna elements and a rough step-size, it does not affect the computational complexity. In addition, Beamspace MUSIC in the proposed cascade algorithm does not degrade the estimation accuracy of signal AOAs compared to using only the Beamspace MUSIC algorithm, because it uses all of the antenna elements and a fine step-size in the specific ranges. Note that in this paper we refer to conventional Beamspace MUSIC as “only Beamspace MUSIC”, to distinguish it from Beamspace MUSIC used in the proposed cascade algorithm. In this paper, a flexible massive antenna is used to select a small number of the total antenna elements, using CAPON.

## 4. Computer Simulation

In this section, we provide computer simulation examples to demonstrate the performance of the proposed cascade AOA estimation algorithm based on the flexible (ON/OFF) massive antenna array. For the simulation, continuous wave (CW), frequency modulation (FM), amplitude modulation (AM), and wideband (WB) noise signals, and AWGN are considered in a received signal, and a flexible antenna array with a size of 10×10 is used as the receiver. In addition, for reliable performance evaluation, we consider four scenarios as follows:

1. The first scenario: three AOA groups (the first AOA group includes one CW, one FM, and one WB noise adjacent signals; the second AOA group includes one AM, one FM adjacent signals; and the third AOA group includes one FM and one AM adjacent signals).
2. The second scenario: two AOA groups (the first AOA group includes two CW, one WB noise adjacent signals; and the second AOA group includes one AM, two WB noise adjacent signals).
3. The third scenario: one AOA group (including two AM, two FM, and one WB noise adjacent signals).
4. The fourth scenario: three AOA groups (the first AOA group includes one CW and one FM signal; the second AOA group includes one AM signal; and the third AOA group includes one FM and one AM adjacent signals).

The primary parameters for the signals considered in the four scenarios are summarized in Table 2, Table 3, Table 4 and Table 5, respectively. Note that we assume that elevation angles of all of the signals for the first, second, and third scenarios are 50°,−20°, and 40°, respectively, for the convenience of the simulation. In the fourth scenario, there are three AOA groups of signals with different elevation angles, unlike in the other three scenarios. Furthermore, we assume that the SNR for each signal is 20 dB, the normalized modulation frequency and the modulation index of the FM signal are $f_{m}=0.001$ and $M.I._{FM}=0.05$, respectively, and the modulation index of the AM signal is $M.I._{AM}=0.03$. To roughly estimate AOA groups employing CAPON, we assume that 4×4 neighboring elements of the antenna are activated, whereas all of the elements (10×10) are activated for estimating individual signal AOAs in the estimated ranges based on AOA groups using Beamspace MUSIC. In the simulation, we consider a 10dB threshold to determine the search ranges of Beamspace MUSIC.

**Figure 4:**
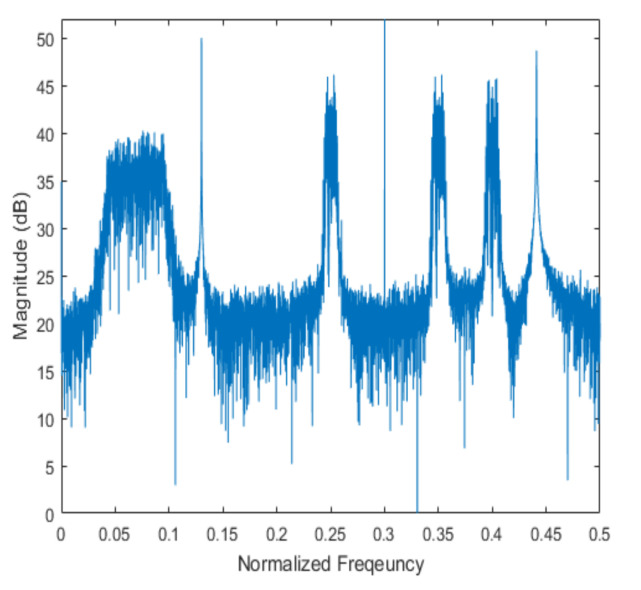
Spectrum of the received signal for the first scenario.

**Figure 5:**
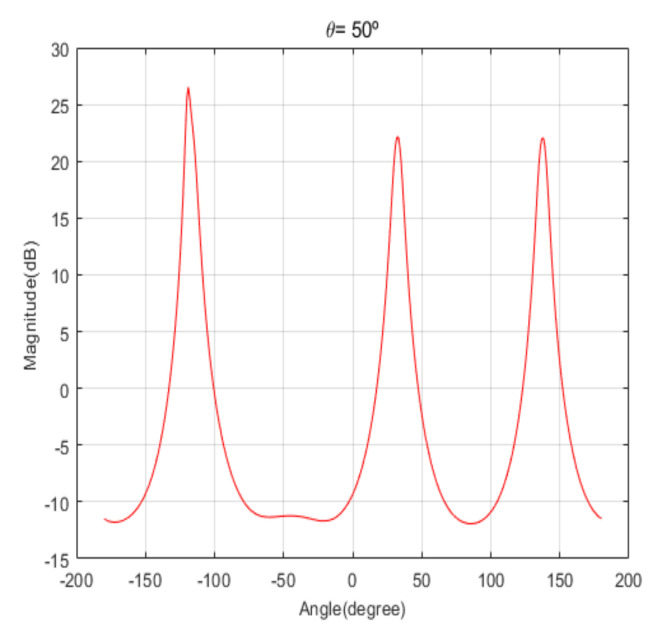
Spatial spectrum of CAPON for the first scenario (elevation angle = 50°).

**Figure 6:**
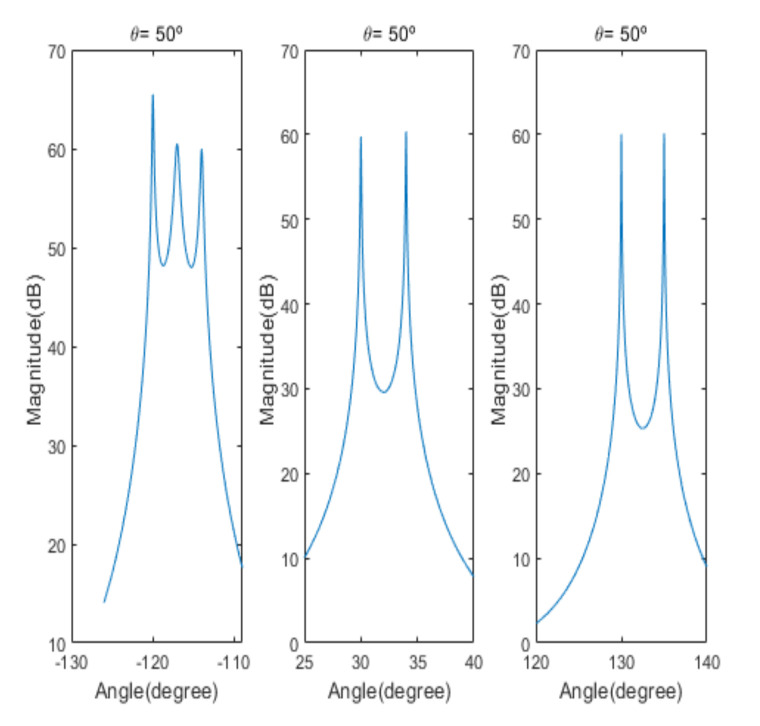
Spatial spectrum of Beamspace Multiple Signal Classification (MUSIC) in the proposed algorithm for the first scenario (elevation angle = 50°).

**Figure 7:**
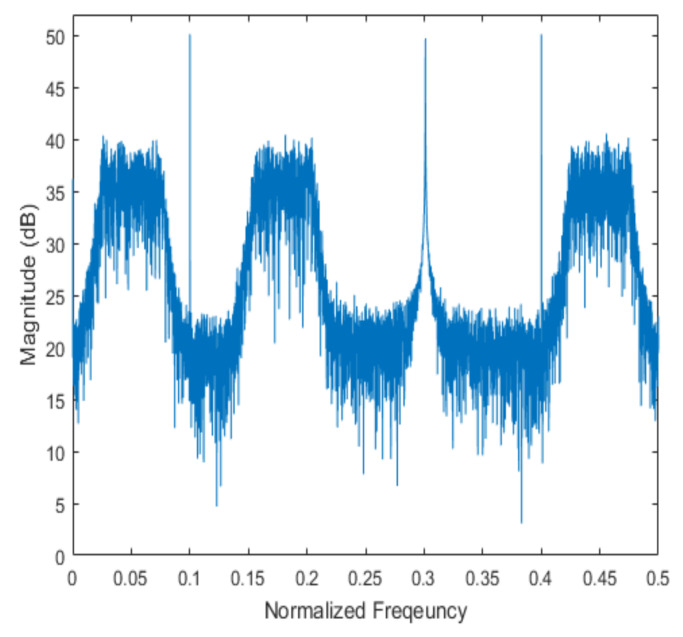
Spectrum of the received signal for the second scenario.

**Figure 8:**
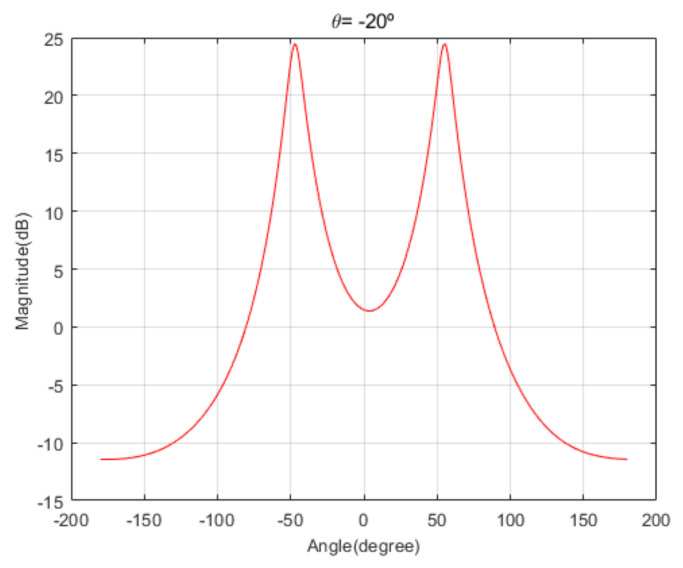
Spatial spectrum of CAPON for the second scenario (elevation angle = −20°).

**Figure 9:**
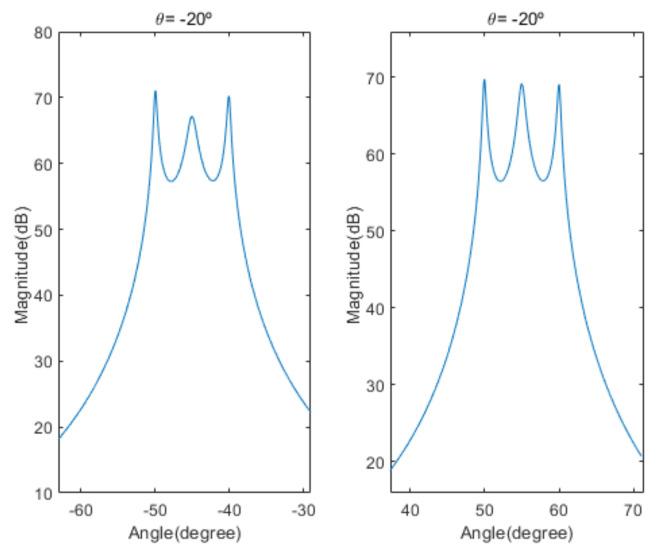
Spatial spectrum of Beamspace MUSIC in the proposed algorithm for the second scenario (elevation angle = −20°).

**Figure 10:**
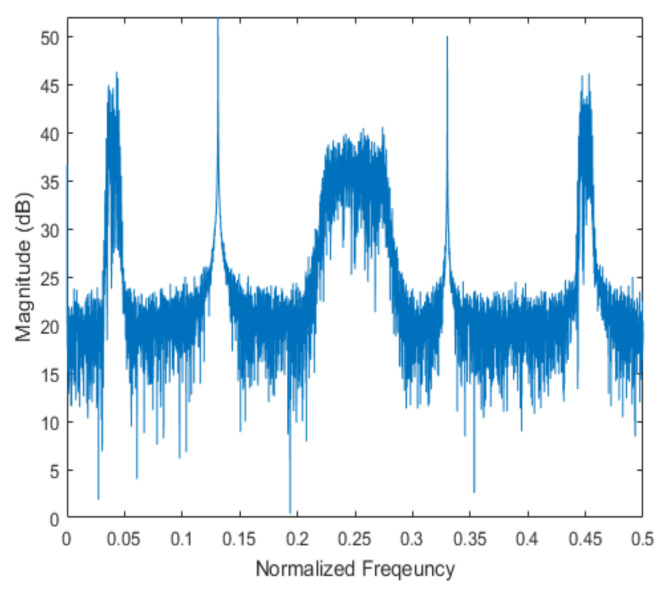
Spectrum of the received signal for the third scenario.

**Figure 11:**
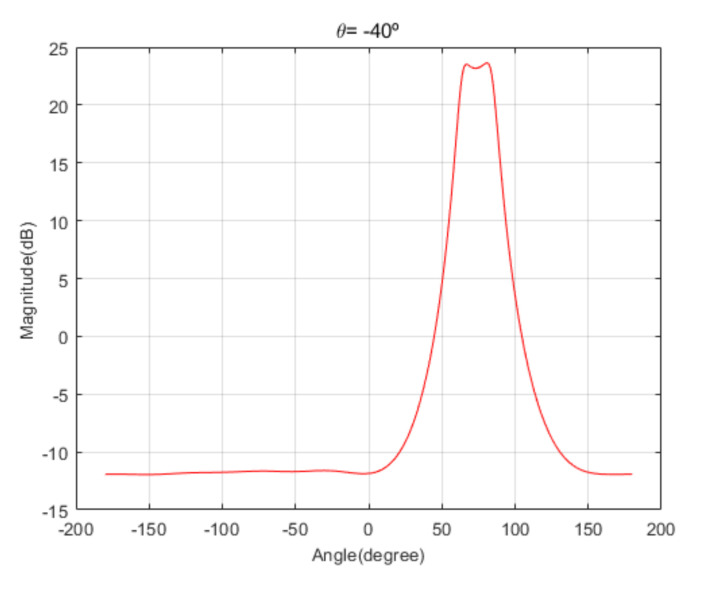
Spatial spectrum of CAPON for the third scenario (elevation angle = −40°).

**Figure 12:**
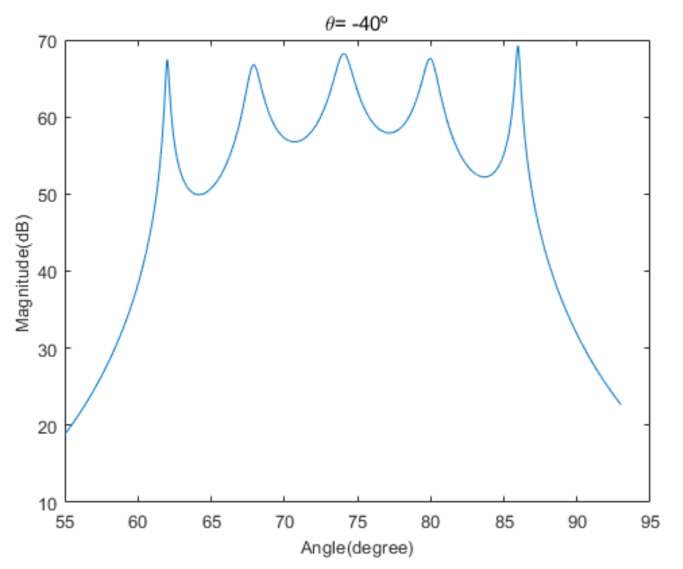
Spatial spectrum of Beamspace MUSIC in the proposed algorithm, for the third scenario (elevation angle = −40°).

**Figure 13:**
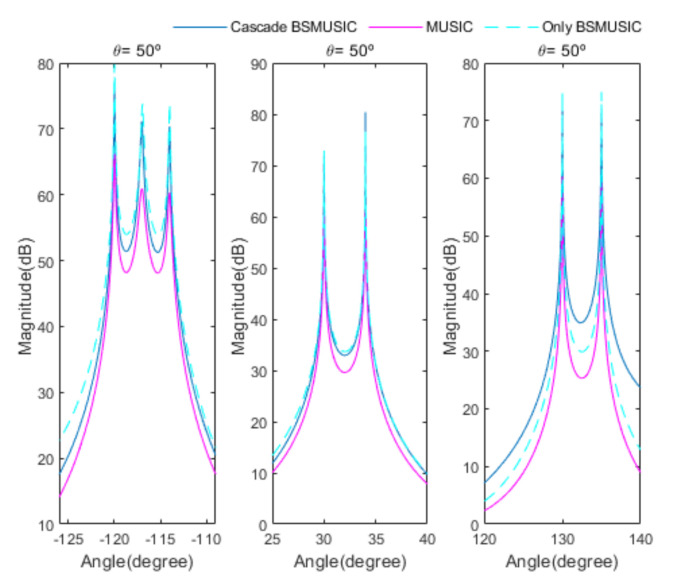
Comparison of the proposed cascade algorithm, MUSIC, and only Beamspace MUSIC, for the first scenario.

**Figure 14:**
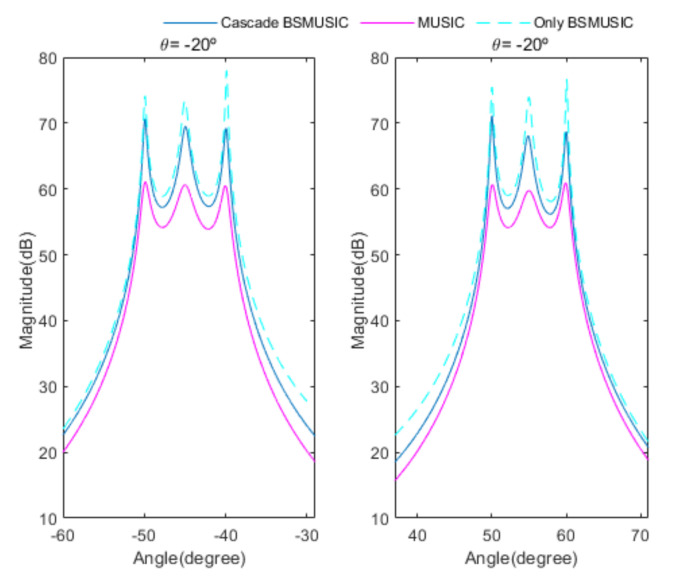
Comparison of the proposed cascade algorithm, MUSIC, and only Beamspace MUSIC, for the second scenario.

**Figure 15:**
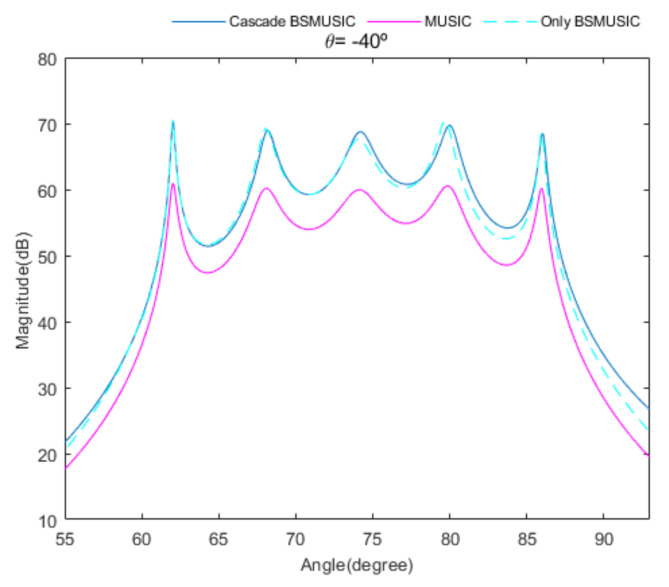
Comparison of the proposed cascade algorithm, MUSIC, and only Beamspace MUSIC, for the third scenario.

**Figure 16:**
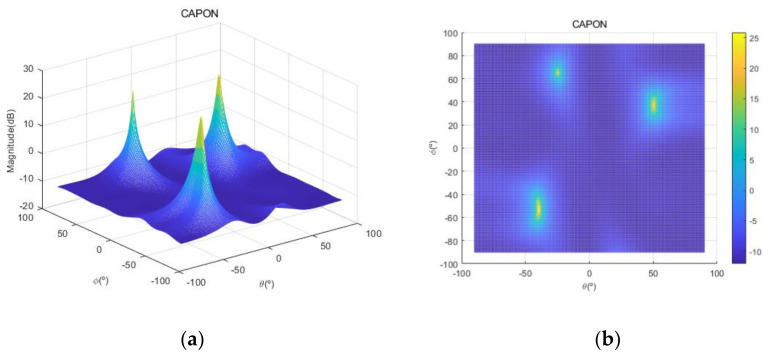
Spatial spectrum of CAPON for the fourth scenario: (**a**) 3D plot, (**b**) top view (θ=−90°~90°,ϕ=−90°~90°).

**Figure 17:**
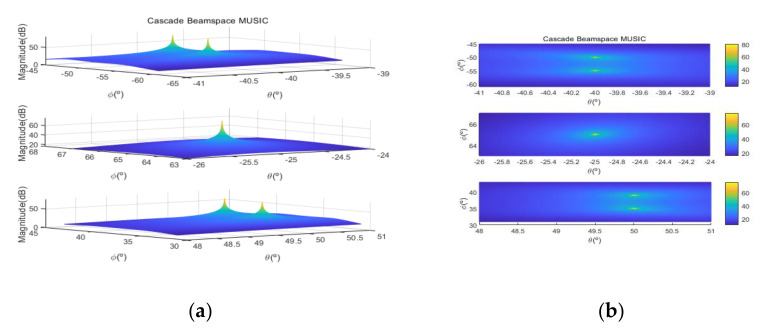
Spatial spectrum of Beamspace MUSIC in the proposed algorithm, for the fourth scenario: (**a**) 3D, (**b**) top view ($\theta_{1}=-41{}^{\circ}\sim -39{}^{\circ},\phi_{1}=-61{}^{\circ}\sim -45{}^{\circ}$,$\theta_{2}=-26{}^{\circ}\sim -24{}^{\circ},\phi_{2}=63{}^{\circ}\sim 67{}^{\circ}$,$\theta_{3}=48{}^{\circ}\sim 51{}^{\circ},\phi_{3}=31{}^{\circ}\sim 43{}^{\circ}$).

**Figure 18:**
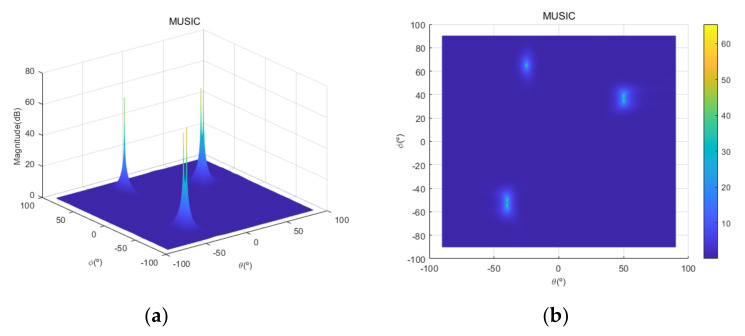
Spatial spectrum of MUSIC for the fourth scenario: (**a**) 3D, (**b**) top view (θ=−90°~90°,ϕ=−90°~90°).

**Figure 19:**
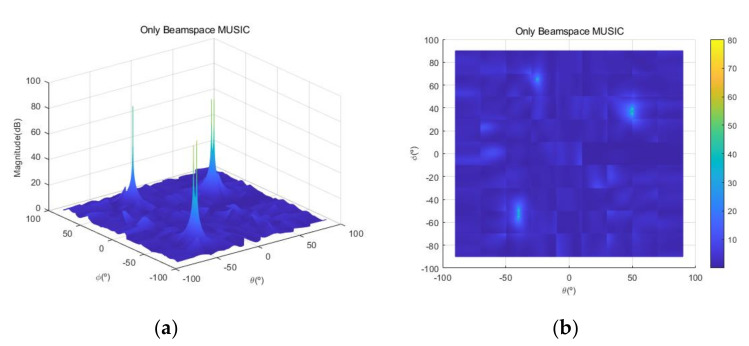
Spatial spectrum of only Beamspace MUSIC for the fourth scenario: (**a**) 3D, (**b**) top view (θ=−90°~90°,ϕ=−90°~90°).

Figure 4 shows the received signal spectrum, including one CW signal, three FM signals, two AM signals, and one WB noise signal, for the first scenario. The spatial spectrum of CAPON based on 4×4 antenna elements for the first scenario, which includes three AOA groups, is shown in Figure 5. Figure 6 presents the spatial spectrum of Beamspace MUSIC based on all of the antenna elements (size of $P_{B}$ is 9) for the first scenario, which shows the final result of the estimated signal AOAs. From Figure 6, we observe that the first AOA group includes three adjacent signal AOAs, the second AOA group includes two signal AOA, and the third AOA group includes two adjacent signal AOAs. Figure 7 shows the received signal spectrum including two CW signals, one AM signal, and three WB noise signals, for the second scenario. The spatial spectrum of CAPON based on 4×4 antenna elements for the second scenario, which includes two AOA groups, is shown in Figure 8. Figure 9 presents the spatial spectrum of Beamspace MUSIC based on all of the antenna elements (size of $P_{B}$ is 9) for the second scenario, which shows the final result of the estimated signal AOAs. From Figure 9, we observe that the first AOA group includes three adjacent signal AOAs and the second AOA group includes three adjacent signal AOAs. Figure 10 shows the received signal spectrum including two FM signals, two AM signals, and one WB noise signal, for the third scenario. The spatial spectrum of CAPON based on 4×4 antenna elements for the third scenario, which includes one AOA group, is shown in Figure 11. Figure 12 presents the spatial spectrum of Beamspace MUSIC based on all of the antenna elements (size of $P_{B}$ is 16) for the third scenario, which shows the final result of the estimated signal AOAs. From Figure 12, we observe that the estimated AOA group includes five adjacent signal AOAs. From Figure 6, Figure 9, and Figure 12, we observe that all signal AOAs are efficiently estimated for all scenarios. Figure 13, Figure 14 and Figure 15 compare the spatial spectra of Beamspace MUSIC of the proposed cascade algorithm to the conventional MUSIC and only Beamspace MUSIC based on DFT. For the performance comparison of three algorithms, we added the results of Figure 6, Figure 9, and Figure 12 into Figure 13, Figure 14 and Figure 15. From the figures, we can see that the estimation performance of the proposed cascade algorithm is similar to those of the conventional MUSIC algorithm and the only Beamspace MUSIC algorithm, because the peak lengths of the three algorithms are similar. Figure 16, Figure 17, Figure 18 and Figure 19 are 3D figures for the fourth scenario considering different azimuth and elevation angles. The 3D spatial spectrum of CAPON based on 4×4 antenna elements for the fourth scenario, which includes three AOA groups, is shown in Figure 16. Figure 17 presents the 3D spatial spectrum of Beamspace MUSIC in the proposed algorithm based on the entire antenna elements for the fourth scenario, which shows the final result of the estimated signal AOAs. From Figure 17, we observe that the first AOA group includes two adjacent signal AOAs, the second AOA group includes one signal AOA, and the third AOA group includes two adjacent signal AOAs. Figure 18 and Figure 19 show the 3D spatial spectra of MUSIC and only Beamspace MUSIC for comparison with the proposed cascade algorithm. From these figures, we observe that the three algorithms have similar AOA estimation performance for the fourth scenario.

**Figure 20:**
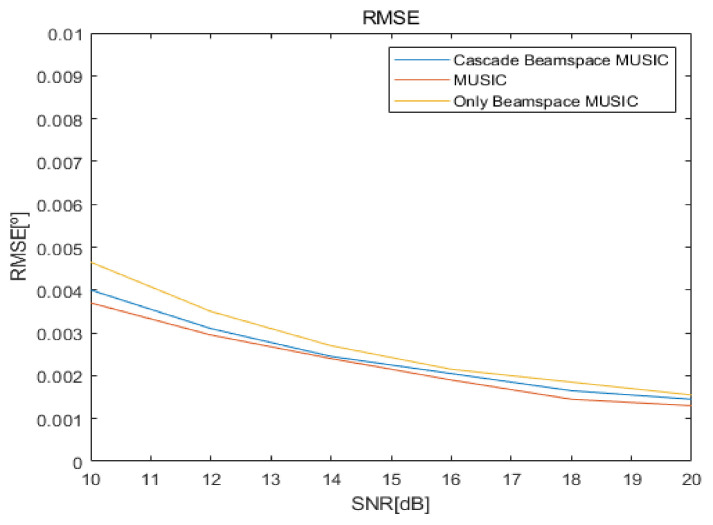
Root mean square error (RMSE) curves according to signal-to-noise ratio (SNR) of proposed cascade, MUSIC, and Beamspace MUSIC.

Figure 20 shows the root mean square error (RMSE) curves versus SNRs for the proposed cascade algorithm, MUSIC, and only Beamspace MUSIC, for the fourth scenario. RMSE is calculated by:

(11) $$RMSE\left(\theta ,\phi \right)=\sqrt{\frac{E\left[{\left(\theta_{o}-\hat{\theta }\right)}^{2}\right]+E\left[{\left(\phi_{o}-\hat{\phi }\right)}^{2}\right]}{2}}$$

where $\theta_{o}$ and $\phi_{o}$ are original elevation and azimuth angles, respectively, and $\hat{\theta }$ and $\hat{\phi }$ are the estimated elevation and azimuth angles, respectively. From Figure 20, we observe that the three algorithms have similar estimation errors for all SNRs for the fourth scenario. We omit RMSE results for other scenarios because they are similar.

## 5. Computational Complexity

In this section, we provide analysis of the computational complexity for the proposed cascade algorithm compared to that of the conventional MUSIC and only Beamspace MUSIC algorithms, to demonstrate the low computational complexity of the proposed approach. Table 6 summarizes the numbers of multiplication/division and addition/subtraction for CAPON, Beamspace MUSIC, and MUSIC, for the specific elevation and azimuth angles. In Table 6, $R_{C}^{-1}$ is the inverse of the CAPON covariance matrix, P is the total number of antenna elements and $P_{C}$ is the number of antenna elements for CAPON, defined in Section 2 and Section 3. In addition, $P_{B}$ and U are the dimension of the beamspace and the number of signals, respectively, for Beamspace MUSIC. For the MUSIC algorithm, T is the total number of signals. The sizes of α and β are $P_{B}\times P_{B}$ and P×P, respectively, in Table 6.

For the convenient comparison of computational complexities for the proposed cascade AOA estimation algorithm and the conventional MUSIC algorithm, we ignore the computational complexity for the generation of covariance matrices and their eigenvalue decomposition processing for the three algorithms, for the following reasons.

1. With the exception of a step of Equation (8), the computational complexity for the generation of the covariance matrix of Beamspace MUSIC, $R_{B}$, is lower than that of the MUSIC algorithm, R, because the size of $R_{B}$ is smaller than that of R.
2. The computational complexity of an eigenvalue decomposition processing for $R_{B}$ is lower than that of R, because the size of $R_{B}$ is smaller than that of R.
3. The computational complexity of eigenvalue decomposition processing for Beamspace MUSIC in the proposed cascade algorithm is lower than or equal to that for the only Beamspace MUSIC algorithm, because the considered search range of the proposed cascade algorithm is smaller than or equal to that of the only Beamspace MUSIC algorithm.
4. The computational complexity for the generation of the covariance matrix of Beamspace MUSIC is higher than that of the general covariance matrix with the same size, due to Equations (8) and (10).
5. We must consider multiple covariance matrices and eigenvalue decompositions of Beamspace MUSIC because we estimate multiple AOA groups in the proposed cascade algorithm and the only Beamspace MUSIC algorithm.
6. The computational complexity for the generation of the covariance matrix of CAPON, $R_{C}$, is significantly lower than that of R, because the size of $R_{C}$ is significantly smaller than that of R.

Based on the aggregation of the above content, we assume that the computational complexities for the generation of the covariance matrix and the eigenvalue decomposition for the proposed cascade algorithm, the general MUSIC algorithm, and the only Beamspace MUSIC algorithm, are similar. From the above content, we find that the computational complexity of the eigenvalue decomposition processing for the general MUSIC algorithm is much higher than that of the other algorithms, and that for the only Beamspace MUSIC algorithm is higher than or equal to that for Beamspace MUSIC in the proposed cascade algorithm. Therefore, we can ignore the computational complexity of the eigenvalue decomposition processing for the three algorithms because the goal of this section is to show that the computational complexity of the proposed cascade algorithm is lower than that of the general MUSIC algorithm and the only Beamspace MUSIC algorithm. In particular, we consider that their computational complexities are lower than those for the calculation of the spatial spectrum of CAPON, Beamspace MUSIC, and general MUSIC, for all of the elevation and azimuth angles. In conclusion, in this section we focus on the comparison of the computational complexities for searching AOAs based on the spatial spectrum of the proposed cascade algorithm, the MUSIC algorithm, and the only Beamspace MUSIC algorithm.

Based on Table 6, the total numbers of the multiplication/division and addition/subtraction for searching AOAs on the spatial spectrum of Beamspace MUSIC in the proposed algorithm are given by:

(12) $$Complexity_{BM}(mul/div)=\sum_{i=1}^{G}\left[\frac{\psi_{\theta_{i}}\psi_{\phi_{i}}}{\delta_{BM}^{2}}\left(P_{B_{i}}^{2}+P_{B_{i}}+1+P_{B_{i}}P\right)+\left(P_{B_{i}}-U_{i}\right)P_{B_{i}}^{2}\right]$$

and:

(13) $$Complexity_{BM}(add/sub)=\sum_{i=1}^{G}\left[\frac{\psi_{\theta_{i}}\psi_{\phi_{i}}}{\delta_{BM}^{2}}\left(P_{B_{i}}^{2}-1+P_{B_{i}}\left(P-1\right)\right)+\left(P_{B_{i}}-U_{i}-1\right)P_{B_{i}}^{2}\right]$$

respectively, where $P_{B_{i}}$ and $U_{i}$ are the dimension of the beamspace and the number of signals, respectively, in the ith estimated groups for Beamspace MUSIC. In addition, G is the number of the estimated AOA groups, $\psi_{\theta_{i}}$ and $\psi_{\phi_{i}}$ are the searching ranges of the ith estimated group, respectively, and $\delta_{BM}$ is the Beamspace MUSIC step-size for searching for AOAs (for example, if the present searching angle is 78° and δ=0.01, the next searching angle is 78.01°). In this calculation, we ignore the computational complexity for generating the beamspace process matrix because its number of multiplication/division and addition/subtraction is very low compared to the that of the other calculations. Considering the computational complexity for CAPON and Beamspace MUSIC, the total numbers of the multiplication/division and addition/subtraction of the proposed cascade AOA estimation algorithm are given by:

(14) $$Complexity_{Cascade}(mul/div)=\frac{360^{2}}{\delta_{C}}\left(P_{C}{}^{2}+P_{C}+1\right)+P_{C}{}^{3}\;+\sum_{i=1}^{G}\left[\frac{\psi_{\theta_{i}}\psi_{\phi_{i}}}{\delta_{BM}^{2}}\left(P_{B_{i}}^{2}+P_{B_{i}}+1+P_{B_{i}}P\right)+\left(P_{B_{i}}-U_{i}\right)P_{B_{i}}^{2}\right]$$

and:

(15) $$Complexity_{Cascade}(add/sub)=\frac{360^{2}}{\delta_{C}}\left(P_{C}{}^{2}-1\right)+P_{C}{}^{3}-2P_{C}{}^{2}+P_{C}\;+\sum_{i=1}^{G}\left[\frac{\psi_{\theta_{i}}\psi_{\phi_{i}}}{\delta_{BM}^{2}}\left(P_{B_{i}}^{2}-1+P_{B_{i}}\left(P-1\right)\right)+\left(P_{B_{i}}-U_{i}-1\right)P_{B_{i}}^{2}\right]$$

respectively, where $\delta_{C}$ is the CAPON step-size for searching for AOAs. In addition, the total numbers of the multiplication/division and addition/subtraction for searching for AOAs on the spatial spectrum of the conventional MUSIC algorithm are given by:

(16) $$Complexity_{M}(mul/div)=\frac{360^{2}}{\delta_{M}^{2}}\left(P^{2}+P+1\right)+\left(P-T\right)P^{2}$$

and:

(17) $$Complexity_{M}(add/sub)=\frac{360^{2}}{\delta_{M}^{2}}\left(P^{2}-1\right)+\left(P-T-1\right)P^{2}$$

respectively, where $\delta_{M}$ is the MUSIC step-size for searching for AOAs. The total number of multiplication/division and addition/subtraction for searching for AOAs using the only Beamspace MUSIC algorithm are given by:

(18) $$Complexity_{OBM}(mul/div)=\frac{360^{2}}{\delta_{BM}^{2}}\left(P_{B}^{2}+P_{B}+1+P_{B}P\right)+G_{OBM}\left(P_{B}-U\right)P_{B}^{2}$$

and:

(19) $$Complexity_{OBM}(add/sub)=\frac{360^{2}}{\delta_{BM}^{2}}\left(P_{B}^{2}-1+P_{B}\left(P-1\right)\right)+G_{OBM}\left(P_{B}-U-1\right)P_{B}^{2}$$

respectively, where $G_{OBM}$ is the number of divided ranges for only Beamspace MUSIC.

**Figure 21:**
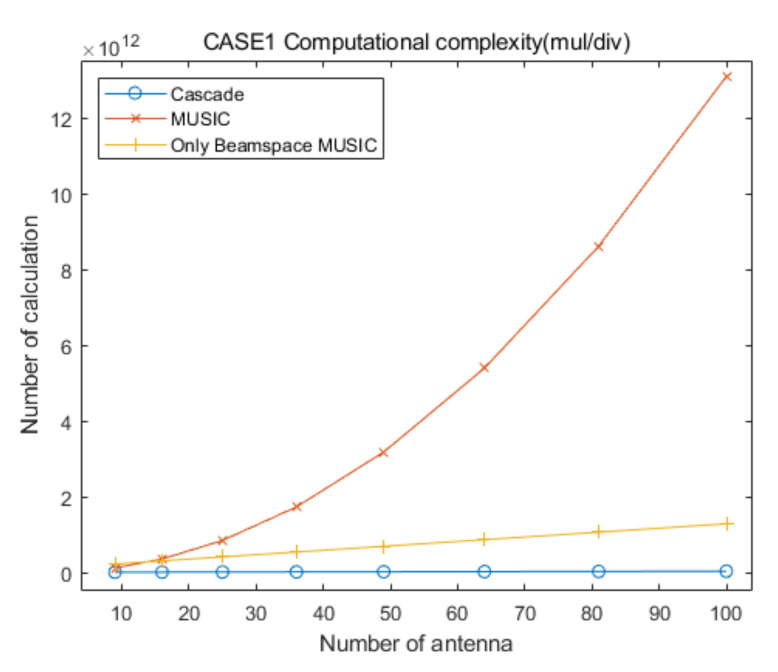
Curves for comparing multiplication/division computational complexities of the proposed cascade algorithm, the general MUSIC algorithm, and only Beamspace MUSIC, versus the number of antenna elements, for CASE 1.

**Figure 22:**
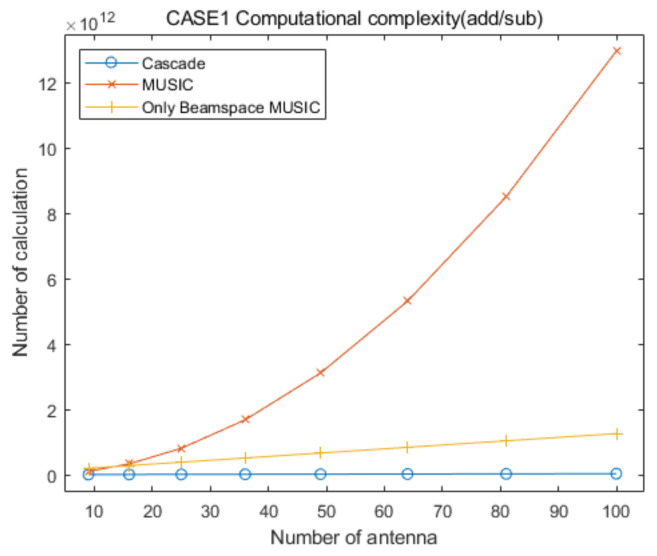
Curves for comparing addition/subtraction computational complexities of the proposed cascade algorithm, the general MUSIC algorithm, and the only Beamspace MUSIC algorithm, versus the number of antenna elements, for CASE 1.

**Figure 23:**
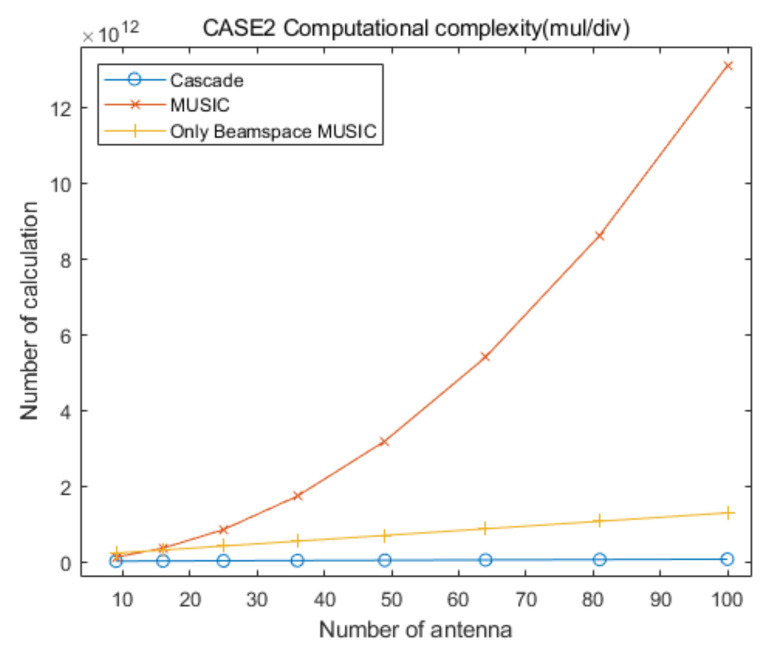
Curves for comparing multiplication/division computational complexities of the proposed cascade algorithm, the general MUSIC algorithm, and only Beamspace MUSIC, versus the number of antenna elements, for CASE 2.

**Figure 24:**
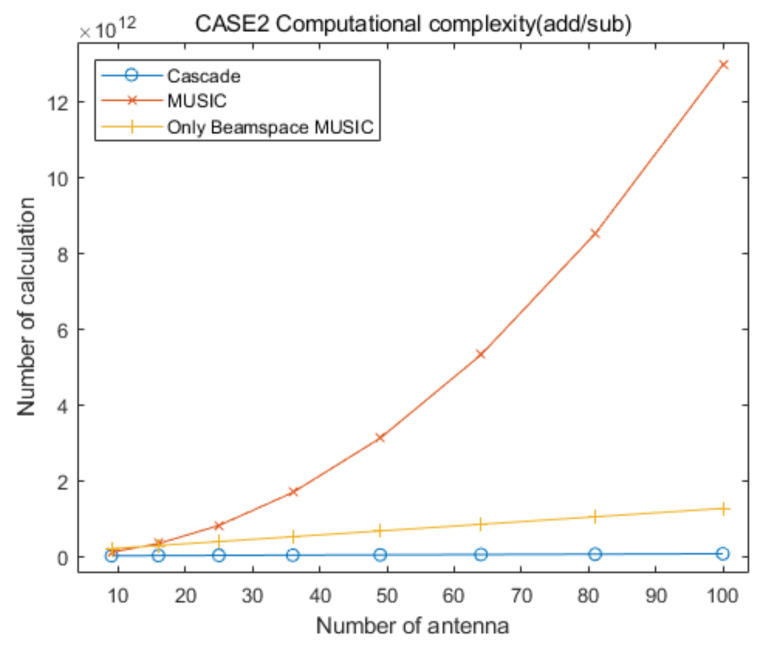
Curves for comparing addition/subtraction computational complexities of the proposed cascade algorithm, the general MUSIC algorithm, versus the number of antenna elements, for CASE 2.

To compare the computational complexities of the proposed cascade algorithm, the general MUSIC algorithm, and the only Beamspace MUSIC algorithm, according to the number of antenna elements, we consider the two cases summarized in Table 7. In addition, we consider square array antennas with sizes from 3×3 to 10×10. Figure 21 and Figure 22 show curves for the numbers of multiplication/division and addition/subtraction versus the number of antenna elements for the proposed cascade algorithm, the general MUSIC algorithm, and the only Beamspace MUSIC algorithm, for CASE 1, respectively. Figure 23 and Figure 24 show curves for the numbers of multiplication/division and addition/subtraction versus the number of antenna elements for the proposed cascade algorithm, the general MUSIC algorithm, and the only Beamspace MUSIC algorithm, for CASE 2, respectively. From these figures, we observe that the computational complexity of the proposed cascade algorithm is lower than that of the general MUSIC algorithm and only Beamspace MUSIC. As the size of the array antenna is increased, the difference in the three curves increases. That is, the proposed cascade algorithm is extremely efficient compared to the general MUSIC algorithm and the only Beamspace MUSIC algorithm for a massive antenna array which has a large number of antenna elements.

**Figure 25:**
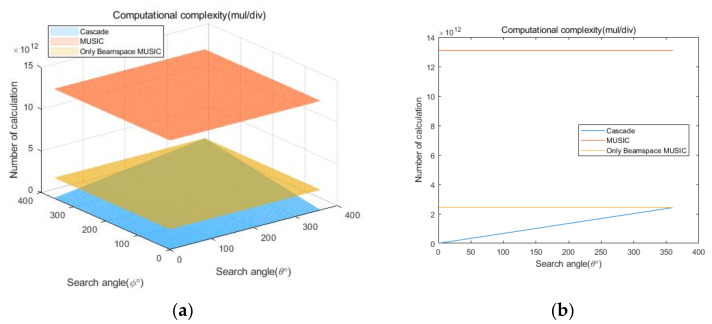
Curves for comparing multiplication/division computational complexities of the proposed cascade algorithm, the general MUSIC algorithm, and only Beamspace MUSIC, according to the search range: (**a**) 3D, (**b**) side view.

**Figure 26:**
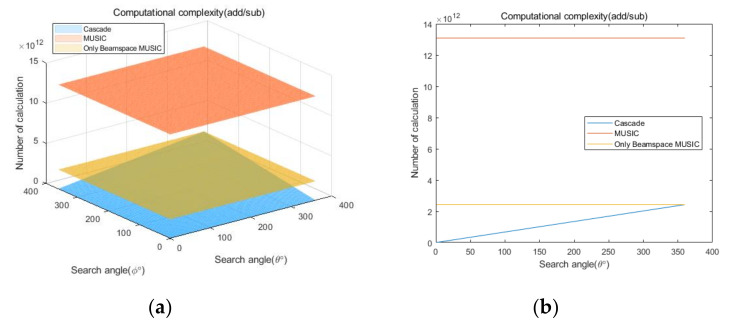
Curves for comparing addition/subtraction computational complexities of the proposed cascade algorithm, the general MUSIC algorithm, and only Beamspace MUSIC, according to the search range: (**a**) 3D, (**b**) side view.

Next, we compare computational complexities of the proposed cascade algorithm, the general MUSIC algorithm, and the only Beamspace MUSIC algorithm, according to the search range. To compare, we assume that the overall antenna size is 10×10, the size of $P_{B}$ is 16, the total number of signals is 16, the number of the AOA groups is 4, and the number of signals in each group is 4. This scenario considers the condition that, for U signals in an AOA group, the high-dimensional array signals can be represented in a 2U-dimensional subspace without information loss for the ULA antenna [44]. Other parameters are the same as those in Table 7. For example, if each range of the AOA group is 10° and the number of the AOA group is 4, the search range of the proposed cascade algorithm is 40°. Although the search range of the general MUSIC and the only Beamspace MUSIC algorithms is 360°, because they must search the entire range, the proposed cascade algorithm has a variable search range. Figure 25 and Figure 26 show curves for the numbers of multiplication/division and addition/subtraction versus the search range for the proposed cascade algorithm, the general MUSIC algorithm, and the only Beamspace MUSIC algorithm. The curves are presented by 3D plots because they should consider elevation and azimuth angles. Figure 25b and Figure 26b represent the side view of the 3D plot to clearly show the difference in the three curves. From the figures, we observe that the computational complexity of the proposed cascade algorithm is significantly lower than that of the general MUSIC algorithm and the only Beamspace MUSIC algorithm. Note that the proposed cascade algorithm is slightly higher than the only Beamspace MUSIC algorithm at 360° due to the computational complexity of the CAPON algorithm. That is, the proposed cascade algorithm is more efficient than the general MUSIC algorithm and the only Beamspace MUSIC algorithm for almost every case.

## 6. Conclusions

To accurately estimate the number of signal AOAs simultaneously, a large antenna array is required in a wireless communication receiver. However, if the size of the antenna elements is increased, the computational complexity for estimating signal AOAs is also dramatically increased for conventional AOA estimation algorithms, such as MUSIC. In this paper, we proposed an efficient cascade AOA estimation algorithm based on a flexible massive antenna, consisting of CAPON and Beamspace MUSIC. First, the proposed cascade algorithm roughly finds AOA groups using CAPON, based on the received signal using a small size of the antenna elements. Next, it estimates the individual signal AOA in the estimated AOA groups using Beamspace MUSIC, based on the received signal using the entire size of antenna elements. To estimate the detailed signal AOA, the Beamspace MUSIC algorithm only searches the ranges estimated from the AOA group, unlike conventional AOA estimation algorithms. The performance of the proposed cascade AOA estimation algorithm was illustrated by computer simulation examples with several scenarios. In addition, we compared and analyzed the computational complexities of the proposed cascade algorithm and the general MUSIC algorithm, for various cases. We are currently studying an adaptive threshold determination technique for efficiently determining the optimized threshold value for the Beamspace MUSIC search range in the proposed cascade AOA estimation algorithm, based on the adaptive signal processing theory. The adaptive threshold research includes a separation technique for overlap caused by two very close groups.

## Figures and Tables

**Table 1:** Summary of cascade AOA estimation algorithm.

Receiving the signal ($r_{C}\left(k\right)$) based on the small size of the antenna array for CAPON Determining G AOA groups including multiple signal AOAs, using CAPON Receiving the signal ($r\left(k\right)$) based on the full size of the antenna array for Beamspace MUSIC Estimating the individual signal AOA in an estimated AOA group using Beamspace MUSIC, in the estimated range Repeat 4 until the Gth group

**Table 2:** Computer simulation scenario 1.

Signal	Elevation (°)	Azimuth (°)	Center Frequency
CW	50	−120	0.3
FM	50, 50, 50	−117, 34, 130	0.25, 0.35, 0.4
AM	50, 50	30, 135	0.13, 0.44
WB	50	−114	0.06

**Table 3:** Computer simulation scenario 2.

Signal	Elevation (°)	Azimuth (°)	Center Frequency
CW	−20, −20	−52, −42	0.1, 0.4
AM	−20	60	0.3
WB	−20, −20, −20	−47, 50, 55	0.05, 0.18, 0.45

**Table 4:** Computer simulation scenario 3.

Signal	Elevation (°)	Azimuth (°)	Center Frequency
AM	−40, −40	−75, −71	0.13, 0.33
WB	−40	−67	0.25
FM	−40, −40	−83, −79	0.04, 0.45

**Table 5:** Computer simulation scenario 4.

Signal	Elevation (°)	Azimuth (°)	Center Frequency
AM	50, −25	39, 65	0.05, 0.13
CW	−40	−55	0.3
FM	−40, 50	−50 35,	0.22, 0.35

**Table 6:** Computational complexity of the three algorithms.

CAPON		
$R_{C}^{-1}$	mul/div	$P_{c}^{3}$
	add/sub	$P_{c}^{3}-2P_{c}^{2}+P_{c}$
$\frac{1}{a_{C}{\left(\theta ,\phi \right)}^{H}R_{C}^{-1}a_{C}(\theta ,\phi )}$	mul/div	$P_{c}{}^{2}+P_{c}+1$
	add/sub	$P_{c}^{2}-1$
**Beamspace MUSIC**		
$\alpha ≜E_{BN}E_{BN}^{H}$	mul/div	$(P_{B}-U)P_{B}{}^{2}$
	add/sub	$(P_{B}-U-1)P_{B}{}^{2}$
$a_{BM}\left(\theta ,\phi \right)$	mul/div	$P_{B}P$
	add/sub	$P_{B}(P-1)$
$\frac{1}{a_{BM}{\left(\theta ,\phi \right)}^{H}\alpha a_{BM}(\theta ,\phi )}$	mul/div	$P_{B}^{2}+P_{B}+1$
	add/sub	$P_{B}^{2}-1$
**MUSIC**		
$\beta ≜E_{N}E_{N}^{H}$	mul/div	$\left(P-T\right)P^{2}$
	add/sub	$\left(P-T-1\right)P^{2}$
$\frac{1}{a{\left(\theta ,\phi \right)}^{H}\beta a(\theta ,\phi )}$	mul/div	$P^{2}+P+1$
	add/sub	$P^{2}-1$

**Table 7:** Scenario for comparing computational complexities of the proposed cascade algorithm, the general MUSIC algorithm, and the only Beamspace MUSIC algorithm.

	CASE 1	CASE 2
The number of total signals (T)	6	8
The number of AOA groups (G)	2	4
The number of signals in AOA group (U)	3	2
Size of $P_{B}$	9	
Search range (CAPON)	360°	360°
Search range (Beamspace MUSIC of proposed cascade algorithm)	40°×G	40°×G
Search range (Only Beamspace MUSIC)	360°	360°
Search range (MUSIC)	360°	360°
Step-size (CAPON)	1°	1°
Step-size (Beamspace MUSIC of proposed cascade algorithm)	0.01°	0.01°
Step-size (Only Beamspace MUSIC)	0.01°	0.01°
Step-size (MUSIC)	0.01°	0.01°

## References

[1] Li J., Halder B., Stoica P., Viberg M. (1995). Computationally efficient angle estimation for signals with known waveforms. IEEE Trans. Signal Process..

[2] Tuncer E., Friedlander B. (2009). Classical and Modern Direction-of-Arrival Estimation.

[3] Krim H., Viberg M. (1996). Two decades of array signal processing research: Parametric approach. IEEE Signal Process. Mag..

[4] Shu F., Qin Y., Liu T., Gui L., Zhang Y., Li J., Han Z. (2018). Low-complexity and high-resolution DOA estimation for hybrid analog and digital massive MIMO receive array. IEEE Trans. Commun..

[5] Godara L.C. (2004). Smart Antennas.

[6] Steinwandt J., De Lamare R.C., Haardt M. (2013). Beamspace direction finding based on the conjugate gradient and the auxiliary vector filtering algorithms. Signal Process..

[7] Foutz J., Spanias A., Banavar M.K. (2008). Narrowband Direction of Arriaval Estimation for Antenna Arrays.

[8] Van Trees H.L. (2002). Optimum Array Processing Part. IV of Detection, Estimation and Modulation Theory.

[9] Gross F. (2005). Smart Antennas for Wireless Communications with Matlab.

[10] Akbari F., Moghaddam S.S., Vakili V.T. MUSIC and MVDR DOA Estimation algorithms with higher resolution and accuracy. Proceedings of the 2010 5th International Symposium on Telecommunications.

[11] Aouina K., Benazzouz D. (2016). 2D-DOA Estimation using split vertical linear and circular arrays. IEEJ Trans. Electr. Electron. Eng..

[12] Liberti J.C., Rappaport T.S. (1999). Smart Antennas for Wireless Communications: IS-95 and Third Generation CDMA Applications.

[13] Wang N., Agathoklis P., Antoniou A. (2006). A new DOA estimation technique based on subarray beamforming. IEEE Trans. Signal Process..

[14] Chen Z., Gokeda G., Yu Y. (2010). Introduction to Direction of Arrival Estimation.

[15] Adam I.A.H., Islam M.R. Performance study of Direction of Arrival (DOA) estimation algorithms for linear array antenna. Proceedings of the 2009 International Conference on Signal Processing Systems.

[16] Lavate T.B., Kokate V.K., Sapkal A.M. Performance analysis of MUSIC and ESPRIT DOA Estimation algorithms for adaptive array smart antenna in mobile communication. Proceedings of the 2010 2nd International Conference on Computer and Network Technology.

[17] Waweru N.P., Konditi D.B.O., Langat P.K. (2014). Performance analysis of MUSIC, root-MUSIC and ESPRIT DOA estimation algorithm. World Acad. Sci. Eng. Technol..

[18] EL-Barbary K.A., Mohamed T.S., Melad M.S. (2013). High resolution direction of arrival estimation (coherent signal source DOA estimation). Int. J. Eng. Res. Appl..

[19] Weber R.J., Huang Y. Analysis for Capon and MUSIC DOA estimation algorithms. Proceedings of the 2009 IEEE Antennas and Propagation Society International Symposium.

[20] Li F., Liu H., Vaccaro R.J. (1993). Performance analysis for DOA estimation algorithms: Unification, simplification, and observations. IEEE Trans. Aerosp. Electron. Syst..

[21] McCloud M.L., Scharf L.L. (2002). A new subspace identification algorithm for high-resolution DOA estimation. IEEE Trans. Antennas Propag..

[22] Li F., Vaccaro R.J. (1992). Sensitivity analysis of DOA estimation algorithms to sensor errors. IEEE Trans. Aerosp. Electron. Syst..

[23] Rao B.D., Hari K.V.S. (1989). Performance analysis of root-MUSIC. IEEE Trans. Acoust. Speech Signal Process..

[24] Baig N.A., Malik M.B. (2013). Comparison of Direction of Arrival (DOA) estimation techniques for closely spaced targets. Int. J. Future Comput. Commun..

[25] Haardt M., Zoltowski M.D., Mathews C.P., Nossek J. 2D Unitary ESPRIT for efficient 2D parameter estimation. Proceedings of the 1995 International Conference on Acoustics, Speech, and Signal Processing.

[26] Yan F., Jin M., Qiao X. (2013). Low-complexity DOA estimation based on compressed MUSIC and its performance analysis. IEEE Trans. Signal Process..

[27] Lee H.B., Wengrovitz M.S. (1990). Resolution threshold of beamspace MUSIC for two closely spaced emitters. IEEE Trans. Acoust. Speech Signal Process..

[28] Buckely K., Xu X.-L. (1990). Spatial-spectrum estimation in a location sector. IEEE Trans. Acoust. Speech Signal Process..

[29] Cao R., Liu B., Gao F., Zhang X. (2017). A Low-Complex One-Snapshot DOA Estimation Algorithm with Massive ULA. IEEE Commun. Lett..

[30] Wang A., Liu L., Zhang J. Low complexity Direction of Arrival (DoA) estimation for 2D massive MIMO systems. Proceedings of the 2012 IEEE Globecom Workshop.

[31] Fukuda W., Abiko T., Nishimura T., Ohgane T., Ogawa Y., Ohwatari Y., Kishiyama Y. Low-complexity detection based on belief propagation in a massive MIMO system. Proceedings of the IEEE 77th Vehicular Technology Conference.

[32] Haghighatshoar S., Caire G. (2018). Low-complexity massive MIMO subspace estimation and tracking from low-dimensional projections. IEEE Trans. Signal Process..

[33] Prabhu H., Edfors O., Rodrigues J., Liu L., Rusek R. A low-complex peak-to-average power reduction scheme for OFDM based massive MIMO systems. Proceedings of the 2014 6th International Symposium on Communications, Control and Signal Processing (ISCCSP).

[34] Shi W., Huang J., Zhang L., Hou Y. The Beamspace Conjugate MUSIC for Non-circular sources. Proceedings of the 2009 4th IEEE Conference on Industrial Electronics and Application.

[35] Capon J. (1969). High resolution frequency-wavenumber spectrum analysis. Proc. IEEE.

[36] Kiong T.S., Salem S.B., Paw J.K.S., Sankar K.P., Darzi S. (2014). Minimum variance distortionless response beamformer with enhanced nulling level control via dynamic mutated artificial immune system. Sci. World J..

[37] Odachi N., Shoki H., Suzuki Y. High-speed DOA estimation using beamspace MUSIC. Proceedings of the 2000 IEEE 51st Vehicular Technology Conference Proceedings.

[38] Mayhan J., Niro L. (1987). Spatial spectral estimation using multiple beam antennas. IEEE Trans. Antennas Propag..

[39] Zoltowski M.D., Kautz G.M., Silverstein S.D. (1933). Beamspace Root-MUSIC. IEEE Trans. Signal Process..

[40] Xu G., Silverstein S.D., Roy R.H., Kailath T. (1994). Beamspace ESPRIT. IEEE Trans. Signal Process..

[41] Yeom D., Park S., Kim J., Lee M. Performance analysis of beamspace MUSIC with beamforming angle. Proceedings of the 2014 8th International Conference on Signal Processing and Communication Systems.

[42] Weiss A.J., Friedlander B. (1994). Preprocessing for direction finding with minimal variance degradation. IEEE Trans. Signal Process..

[43] Yuri N., Ilia P. Performance study of beamspace processing DOA estimation by MUSIC and capon methods. Proceedings of the 2015 International Siberian Conference on Control and Communications.

[44] Zhao H., Zhang N., Shen Y. (2002). Beamspace direct localization for large-scale antenna array systems. IEEE Trans. Signal Process..

